# Composite Compensation Method for Scale-Factor Nonlinearity in MEMS Gyroscopes Based on Initial Calibration

**DOI:** 10.3390/mi16080851

**Published:** 2025-07-24

**Authors:** Zhaoyin Ding, Yi Zhou

**Affiliations:** 1 Faculty of Engineering, Anhui Sanlian University, Hefei 230601, China; dingzhaoyin2025@163.com; 2 School of Mechanical Engineering, Nanjing University of Science and Technology, Nanjing 210094, China

**Keywords:** MEMS gyroscope, scale factor nonlinearity, initial calibration, repeatability, composite compensation

## Abstract

With the advancement of error correction techniques such as quadrature suppression and mode matching, the bias stability and overall accuracy of MEMS gyroscopes have been greatly improved. However, scale-factor nonlinearity often being underestimated has emerged as a critical barrier to further performance enhancement in high-precision MEMS gyroscopes. This study investigates the mechanism of scale-factor nonlinearity in closed-loop MEMS gyroscopes and introduces the concept of scale-factor repeatability error. A constraint relationship between scale-factor nonlinearity and repeatability is analytically established. Based on this insight, a composite compensation method incorporating initial calibration is proposed to enhance scale-factor linearity. By improving repeatability, the effectiveness and accuracy of polynomial fitting-based compensation are significantly improved. Experimental results show that the proposed method reduces the scale-factor nonlinearity error from 2232.039 ppm to 99.085 ppm, achieving a 22.5-fold improvement. The proposed method is also applicable to other MEMS gyroscopes with similar architectures and control strategies.

## 1. Introduction

Microelectromechanical system (MEMS) gyroscopes are micro-inertial sensors fabricated using silicon-based MEMS technology and operate based on the Coriolis effect [1]. By leveraging the combined advantages of silicon materials and MEMS processes, these devices feature compact size, low weight, high reliability, and favorable dynamic performance, making them highly promising for both military applications, such as guided munitions and tactical weapons, and civilian fields, including vehicle navigation and medical instrumentation [2]. With the rapid growth in demand for high-dynamic-platform motion control and complex environmental perception, the performance requirements for MEMS gyroscopes have become increasingly stringent in order to meet the challenges posed by more demanding application scenarios [3,4].

As a key indicator of a gyroscope’s dynamic performance, the stability of the scale factor directly affects measurement accuracy. For instance, under an input angular rate of 10°/s, a 1000 ppm scale-factor error results in a measurement error of 0.03°/s, which is intolerable for high-precision gyroscopes [5,6]. In general, scale-factor nonlinearity is primarily influenced by factors such as structural design, fabrication precision, and signal processing circuitry. Therefore, improvements in scale-factor performance are typically pursued from these perspectives. To mitigate scale-factor nonlinearity, in 2013, Igor P. Prikhodko et al. from the University of California proposed a self-compensation method based on the temperature dependence of the gyroscope’s resonant frequency. This approach reduced the scale-factor nonlinearity to 700 ppm [7]. In 2014, researchers from the National Aviation University of Ukraine introduced a multi-parameter compensation scheme for gyroscope scale-factor errors [8]. By establishing a polynomial model relating the scale factor to parameters such as input angular rate, resonant frequency, and drive amplitude, the proposed method effectively compensated for the scale-factor error and achieved a reduction of approximately 60%. In 2017, the University of Michigan proposed an on-chip micro-calibration platform for scale-factor calibration in MEMS gyroscopes [9]. This method enabled correction of scale-factor errors with instability of less than 30 ppm. In 2018, the Georgia Institute of Technology introduced a dual-mode drive-and-sense scheme for axisymmetric gyroscope structures [10]. By employing virtual Coriolis-force-based compensation, the scale-factor error was reduced to 62 ppm. In 2019, Daryosh Vatanparvar et al. from the University of California investigated the impact of frequency splitting on scale-factor performance and demonstrated that mode coupling is a primary source of scale-factor nonlinearity. Their method achieved a 27.7% reduction in scale-factor error under a 1 Hz angular rate input [11].

In summary, existing techniques for compensating gyroscope scale-factor errors can be broadly classified into four categories: algorithm-based compensation, virtual Coriolis force self-calibration, micro-vibration platform calibration, and frequency splitting suppression. Algorithmic approaches such as polynomial fitting [12], neural networks [13], fuzzy logic [14], and data fusion methods [15] are relatively easy to implement and cost-effective. However, these methods often neglect the impact of scale-factor repeatability, which limits their robustness and generalization across varying conditions. Virtual Coriolis force self-calibration techniques [16,17,18] enable automatic compensation by exciting the sense mode; however, the additional mechanical vibrations introduced can restrict the gyroscope’s dynamic range and long-term stability. Calibration using micro-vibration platforms [9,19,20] offers high precision but entails substantial cost and complexity, making it less suitable for extreme or resource-constrained environments. Meanwhile, techniques aimed at minimizing frequency splitting or suppressing quadrature errors [21] can improve the scale factor’s linearity in open-loop systems. However, under force-to-rebalance (FTR) operation, the influence of frequency mismatch and quadrature errors on scale-factor nonlinearity becomes negligible. Theoretically, the closed-loop scale factor in FTR mode is decoupled from these error sources, further limiting the effectiveness of such methods in this context.

To overcome the limitations of conventional algorithm-based compensation methods, this study presents a novel approach that investigates the intrinsic mechanism of scale-factor nonlinearity while introducing the concept of scale-factor repeatability as a critical factor influencing compensation accuracy. A theoretical analysis is conducted to quantify the impact of repeatability on compensation performance, highlighting the necessity of improving repeatability to ensure robust and accurate correction. Building on these insights, a composite compensation strategy is proposed that combines initial calibration to enhance repeatability with a piecewise polynomial fitting technique for effective nonlinear error compensation. Experimental validation demonstrates that the proposed method reduces scale-factor nonlinearity from 2232.039 ppm to 99.085 ppm, achieving a 22.5-fold improvement. These results confirm the method’s effectiveness and practical applicability, offering a robust and scalable solution for high-precision MEMS gyroscope compensation.

## 2. Mechanical Structure of MEMS Gyroscopes

### 2.1. Mechanical Structure of the QMG

**Figure 1:**
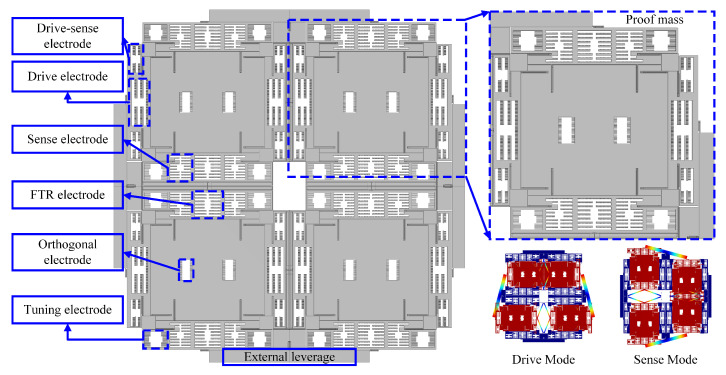
Mechanical structure and operating modes of the MEMS QMG.

The MEMS Quad-Mass Gyroscope (QMG) features a fully symmetric structure and operating modes. It combines a large effective mass with the capability to effectively suppress linear acceleration and other common-mode interferences. Therefore, this paper selects the QMG as the experimental subject. The overall structure of the QMG is symmetric about the drive (X) and sense (Y) axes in both horizontal and vertical directions. It consists of four identical sensing proof masses, each connected to the drive and sense axes via supporting springs in the respective directions. Furthermore, through internal coupling mechanisms and external levers, the four proof masses execute anti-phase motions in both the drive and sense modes, forming a dual-differential drive and dual-differential sense configuration. The gyroscope operates primarily in the first-order modes corresponding to these two modal directions, which move in anti-phase to each other to avoid interference from low-frequency modes, thereby suppressing disturbances from linear acceleration and environmental vibrations. The sensing mode employs large capacitance comb electrodes and a differential sensing mechanism to enhance mechanical sensitivity while reducing common-mode interference. The mechanical structure and operating modes of the QMG are illustrated in Figure 1.

### 2.2. Dynamic Model

**Figure 2:**
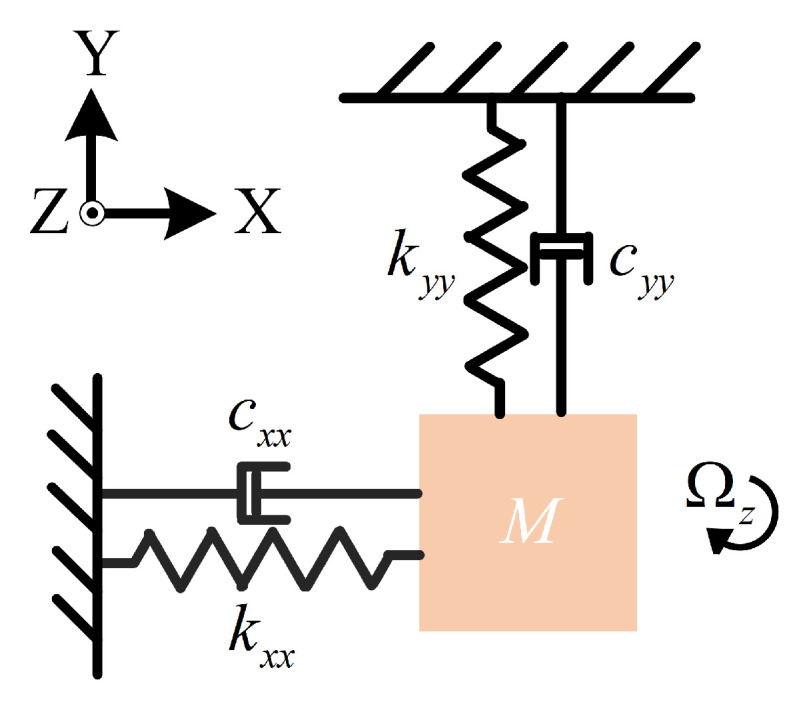
Simplified dynamic model of the MEMS QMG.

The MEMS quad mass gyroscope (QMG) investigated in this work operates in the first-order vibratory mode, with two working modes: the drive mode (X-axis) and the sense mode (Y-axis). Under ideal conditions, the motions of the X and Y modes are completely decoupled. The ideal dynamic behavior of the system can be modeled as a second-order mass–spring–damper system. A simplified schematic of the equivalent dynamic model is shown in Figure 2. In this model, the X-axis and Y-axis are assumed to be perfectly orthogonal, functioning as the drive and sense axes, respectively.

Considering fabrication errors, the dynamic model of the MEMS quad-mass gyroscope can be expressed as in Equation (1):

(1) $$\begin{array}{cc} \; & M\ddot{q}+D\dot{q}+Kq+2mA_{g}\Lambda \dot{q}=F\\ \; & M=\left[\begin{array}{cc} m_{x} & 0\\ 0 & m_{y} \end{array}\right],\;D=\left[\begin{array}{cc} c_{xx} & c_{xy}\\ c_{yx} & c_{yy} \end{array}\right],\\ \; & K=\left[\begin{array}{cc} k_{xx} & k_{xy}\\ k_{yx} & k_{yy} \end{array}\right],\;\Lambda =\left[\begin{array}{cc} 0 & -\Omega_{z}\\ \Omega_{z} & 0 \end{array}\right] \end{array}$$

where $q={\left[x,y\right]}^{T}$ denote the vibration displacements of the drive and sense modes, respectively, $q={\left[F_{x},F_{y}\right]}^{T}$ represent the electrostatic forces applied to the drive and sense modes, $m_{x}=m_{y}$ are the equivalent masses of the drive and sense modes, $c_{xx}$ and $c_{yy}$ are the equivalent damping coefficients, $k_{xx}$ and $k_{yy}$ are the equivalent stiffness coefficients for the drive and sense modes, respectively, $A_{g}$ is the angular gain, representing the proportion of the sensitive mass that provides the Coriolis force, $\Omega_{z}$ is the input angular rate to which the gyroscope is sensitive, and $c_{xy}=c_{yx}$ are the damping and stiffness coupling coefficients between the drive and sense modes. Table 1 lists the structural parameters of the MEMS quad-mass gyroscope.

The parameters listed in Table 1 are derived from the design specifications. The equivalent mass of the gyroscope is 1.56 mg. The resonant frequency and quality factor of the drive mode (X-axis) are 4634 Hz and $1.02\times 10^{6}$, respectively. For the sense mode (Y-axis), the resonant frequency is 4711 Hz, and the quality factor is $0.34\times 10^{6}$, resulting in a frequency split of 77 Hz between the two modes. The velocity-to-current transduction factor for the drive-mode sensing comb is $2.41\times 10^{-6}$ A/(m/s), and the voltage-to-force conversion factor of the drive comb electrodes is $2.79\times 10^{-9}$ N/V. For the sense mode, the corresponding velocity-to-current factor is $6.61\times 10^{-6}$ A/(m/s), and the voltage-to-force coefficient is $4.52\times 10^{-9}$ N/V. The gyroscope die is wire bonded to a metal housing using gold wires, with overall dimensions of $16\times 16\times 3\;{\mathrm{mm}}^{3}$. These details have also been supplemented in the manuscript.

## 3. Analysis of Closed-Loop Scale-Factor Nonlinearity Mechanism

This chapter begins with the closed-loop scale-factor expression of the gyroscope and analyzes the impact of relevant parameters on the nonlinearity of the closed-loop scale factor. Additionally, following the principle of the control variable method, the deviation caused by each individual parameter on the closed-loop scale-factor nonlinearity is investigated to quantify its influence. This analysis lays the foundation for the composite compensation method for the closed-loop scale-factor nonlinearity error proposed in this paper.

### 3.1. Analysis of Nonlinearity Induced by Drive Frequency

When the gyroscope operates in force-to-rebalance (FTR) mode, the closed-loop scale factor of the gyroscope, $SF_{close}$, can be expressed as in Equation (2):

(2) $$SF_{close}=\frac{-2A_{g}m_{y}A_{x}\omega_{d}}{K_{vf}}$$

where $A_{x}$ is the vibration displacement amplitude of the drive mode, $\omega_{d}$ is the drive frequency of the gyroscope’s drive mode, $K_{vf}=4n\varepsilon_{0}hV_{dc}/d_{0}$ is the voltage-to-electrostatic force conversion coefficient, $\varepsilon_{0}$ is the vacuum permittivity, *h* is the thickness of the comb capacitance, $d_{0}$ is the pitch between the comb teeth, *n* is the number of movable comb fingers, and $V_{dc}$ is the DC bias voltage signal, generated by a reference voltage source and applied to the gyroscope’s excitation electrodes. From Equation (2), it can be observed that since the equivalent mass and drive amplitude of the gyroscope generally do not change with the angular rate, the closed-loop scale factor is primarily influenced by the drive frequency signal and the voltage-to-electrostatic force conversion stage. When the gyroscope operates normally, $\omega_{d}=\omega_{x}$. Combining this condition with the dynamic equation of the gyroscope (Equation (1)), the relationship between the drive mode resonant frequency and the input angular rate can be expressed as in Equation (3):

(3) $$\omega_{x}\left(\Omega \right)=\sqrt{\omega_{x}^{2}-\Omega_{z}^{2}}$$

Then, when the input angular rate is $\Omega_{z}$, the scale-factor deviation caused by the drive resonant frequency is given by

(4) $$E_{\omega_{x}}\left(\Omega \right)=\frac{2A_{g}m_{y}A_{x}}{K_{vf}}\left|\frac{\omega_{x}\left(0\right)-\omega_{x}\left(\Omega \right)}{\omega_{x}\left(0\right)}\right|$$

**Figure 3:**
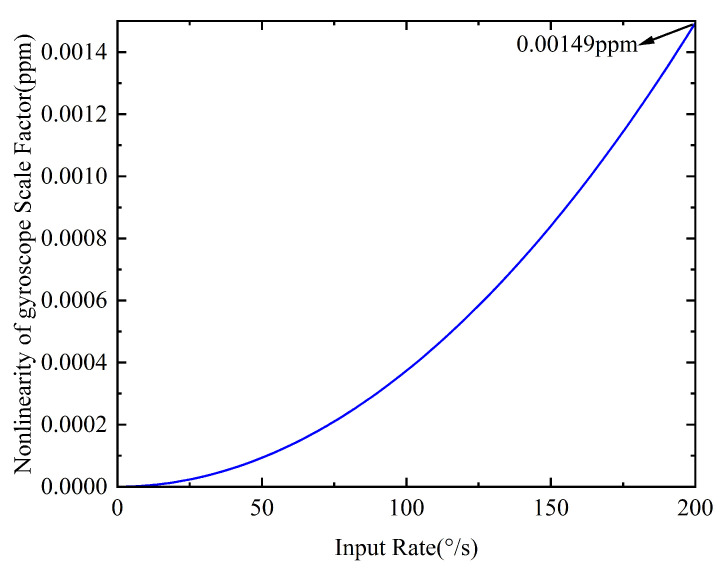
Closed-loop scale-factor deviation induced by the drive frequency.

By substituting the drive mode resonant frequency from Table 1 into Equation (4), the relationship between the scale-factor deviation $E_{\omega_{x}}\left(\Omega \right)$ and the input angular rate $\Omega_{z}$ is obtained, as shown in Figure 3.

As shown in Figure 3, when the input angular rate is 200°/s, the scale-factor deviation $E_{\omega_{x}}\left(\Omega \right)$ is only 0.00149 ppm, indicating that in force-to-rebalance mode, the closed-loop scale-factor nonlinearity is hardly affected by the drive frequency. However, it is worth noting that due to the temperature characteristics of silicon micro-materials, temperature variations within the gyroscope’s internal structure during the actual operation cause resonant frequency drift in both the drive and sense modes. This degrades the repeatability of the closed-loop scale factor and often leads to the underperformance of polynomial fitting-based scale-factor nonlinearity compensation methods.

### 3.2. Analysis of Nonlinearity Introduced by the Sensing Force-Feedback Comb Electrode

**Figure 4:**
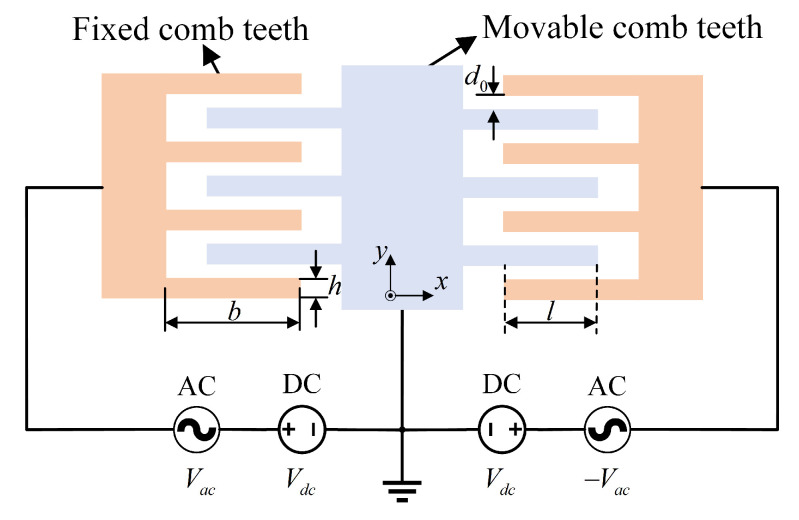
Schematic diagram of the FTR comb capacitor structure.

The voltage-to-electrostatic force conversion stage includes the sense-mode force-feedback comb electrodes and the force-feedback voltage-generation circuit. The voltage-to-electrostatic force conversion in the sense mode is realized through the FTR comb electrodes, whose structure is consistent with the sense-mode drive comb capacitance structure, as illustrated in Figure 4.

The capacitance of a single movable FTR comb finger *C* is given by

(5) $$C=\frac{2n\varepsilon_{0}hl}{d_{0}}+\frac{\left(2n+1\right)\varepsilon_{0}hb}{d_{0}}$$

The motion direction of the sense force-feedback comb fingers is along the y-axis. When a voltage *V* is applied between the comb fingers, the resulting electrostatic force *F* can be expressed as

(6) $$F=\frac{1}{2}\frac{\partial C}{\partial y}V^{2}=\frac{1}{2}\left[\frac{2n\varepsilon_{0}hl}{d_{0}}+\frac{\left(2n+1\right)\varepsilon_{0}hb}{{\left(d_{0}-y\right)}^{2}}\right]V^{2}$$

However, in the actual circuit, the voltage *V* is a combined AC and DC voltage $V_{dc}+V_{ac}sin\left(\omega_{d}t\right)$, and the force-feedback comb fingers are driven by a push–pull dual drive. Therefore, the total electrostatic force of the force-feedback comb fingers can be expressed as

(7) $$F_{y}=\left[\frac{4n\varepsilon_{0}hl}{d_{0}}+\frac{2\left(2n+1\right)\varepsilon_{0}hb}{{\left(d_{0}-y\right)}^{2}}\right]V_{dc}V_{ac}\sin\left(\omega_{d}t\right)$$

Since the DC bias voltage is constant, it can be observed that the first term in Equation (7) represents an electrostatic force linearly related to the amplitude of the AC voltage, while the second term is a nonlinear component that may introduce nonlinearity. However, when the gyroscope operates in closed-loop force-feedback mode, the sense mode is maintained at its equilibrium position due to the combined effects of the Coriolis force and feedback force, resulting in zero vibration displacement. Therefore, the voltage-to-electrostatic force conversion can be considered linear, and the voltage-to-electrostatic force gain does not vary with the input angular rate. Consequently, the sensing force-feedback comb electrodes do not introduce nonlinearity.

### 3.3. Analysis of Nonlinearity Introduced by the Force-Feedback Voltage-Generation Circuit

**Figure 5:**
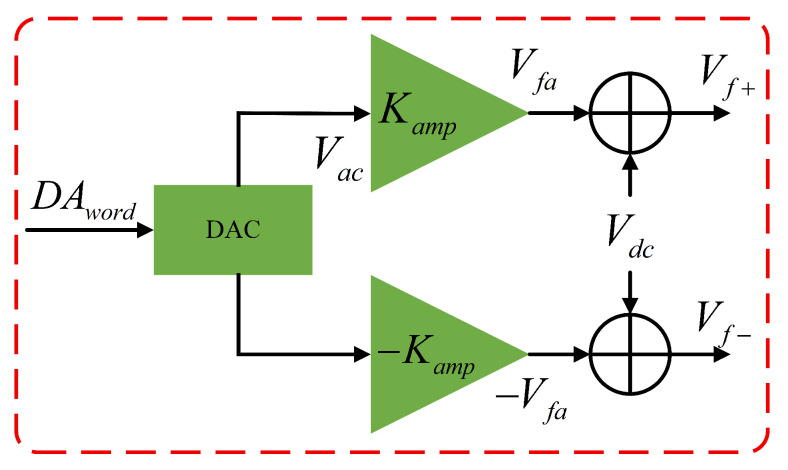
Differential excitation circuit for FTR based on fixed DC-to-AC modulation.

When the force-to-rebalance (FTR) rate measurement control loop adopts a fixed DC-to-AC differential excitation method, the output of the feedback controller must be modulated by a carrier signal within the FPGA. After being output through the DAC, it passes through an AC/DC coupling circuit to generate the FTR voltage. The FTR differential excitation circuit is shown in Figure 5.

Where $DA_{word}$ is the DAC control word and $K_{a}$ is the gain of the non-inverting amplifier. The AC signal $V_{ac}$ output from the DAC, $V_{fa}$ is amplified by the non-inverting amplifier to generate the AC component of the FTR voltage. Additionally, an inverting amplifier with a gain of −1 produces signals. The signals $V_{f}a$ and $-V_{f}a$ are then coupled with the DC bias voltage $V_{dc}$ through AC/DC coupling modules to obtain the FTR voltages $V_{f+}$ and $V_{f-}$, respectively.

However, in the FTR differential excitation circuit, limitations in the DAC chip, such as resolution, update rate, and differential nonlinearity (DNL), may introduce nonlinear distortion in the output signal. To verify this hypothesis, an experiment was conducted to independently evaluate the nonlinearity of the force-feedback voltage-generation circuit. Under ideal conditions, the input angular rate and the DAC control should exhibit a linear relationship. By using the DAC to simulate the ideal AC feedback voltage corresponding to different angular rates, the actual DAC output can be measured and fitted to obtain the relationship curve between the generated feedback voltage and the input angular rate. This curve serves to validate the nonlinearity introduced by the voltage-generation circuit. The test results are summarized in Table 2.

In the actual rate table experiment, the measured scale-factor nonlinearity was 539.281 ppm. According to the data in Table 2, the nonlinearity of the AC signal generated by the DAC was calculated to be 460.679 ppm, accounting for 85.424% of the total closed-loop scale-factor nonlinearity.

The equivalent input angular rate was obtained by fitting a linear curve to the measured data, which is theoretically expected to be linear. However, the output signal after DAC conversion exhibited significant nonlinearity. Therefore, it can be concluded that the DAC conversion stage in the FTR differential excitation circuit is the primary source of scale-factor nonlinearity under the fixed DC-to-AC excitation method. Unfortunately, this type of nonlinearity is often difficult to mitigate through device selection or hardware optimization alone.

## 4. Composite Compensation Method with Initial Calibration

In the previous section, the nonlinearity mechanism of the gyroscope’s closed-loop scale factor was analyzed, and it was established that the primary source of nonlinearity arises from the force-feedback voltage-generation circuit. This type of nonlinearity is difficult to mitigate through device selection or hardware optimization. To address this issue, a composite compensation method with initial calibration is proposed in this paper. By performing initial calibration of the scale factor, the impact of scale-factor repeatability errors on the accuracy of nonlinear compensation can be reduced, thereby improving the overall compensation effectiveness for scale-factor nonlinearity.

### 4.1. Initial Calibration Method for Scale Factor

**Figure 6:**
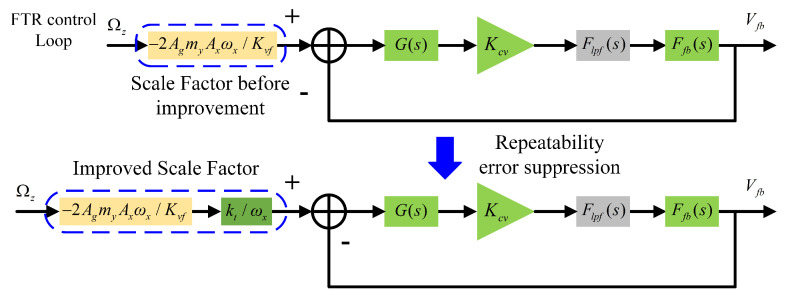
Proposed initial calibration approach for the closed-loop scale factor.

As discussed in Section 3.1, the closed-loop scale factor is inevitably affected by the resonant frequency drift of the drive mode, resulting in significant deviations in the measured scale factor across different trials. Therefore, it is necessary to perform initial calibration of the gyroscope’s closed-loop scale factor to suppress repeatability errors. Figure 6 illustrates the initial calibration method proposed in this paper. After repeatability error suppression, the expression of the closed-loop scale factor can be represented as in Equation (8):

(8) $$SF_{new}=SF_{close}\times \frac{k_{t}}{\omega_{x}}=\frac{-2A_{g}m_{y}A_{x}k_{t}}{K_{vf}}$$

where $k_{t}$ is a constant used to maintain the order of magnitude of the scale factor. As shown in Equation (8), the calibrated closed-loop scale factor is no longer affected by the drive mode resonant frequency, thereby effectively reducing the repeatability error of the closed-loop scale factor.

### 4.2. Piecewise Scale-Factor Nonlinearity Compensation Method

**Figure 7:**
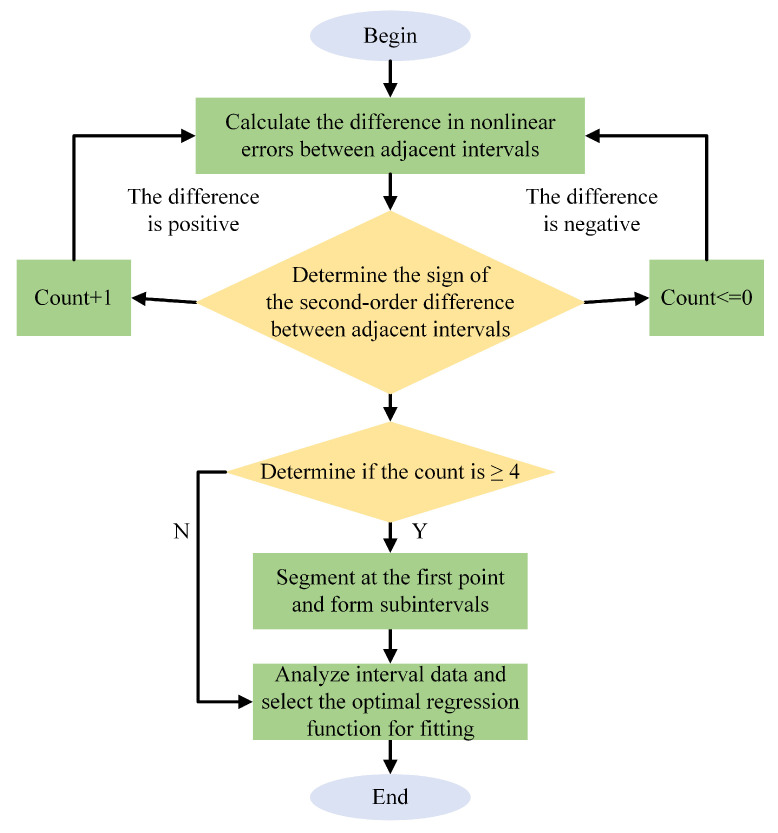
Flowchart of the piecewise scale factor.

Figure 7 illustrates the flowchart of the piecewise compensation method for the gyroscope’s closed-loop scale factor, which consists of the following three main steps:

Step 1: Starting from the static state, the actual output data of the gyroscope are measured at angular rates of ±0.1°/s, ±0.2°/s, ±0.5°/s, ±1°/s, ±2°/s, ±5°/s, ±10°/s, ±20°/s, ±50°/s, ±100°/s, and ±200°/s. The nonlinear errors at each angular rate are then calculated based on the ideal gyroscope output values.

Step 2: The absolute differences between the nonlinear error values of adjacent intervals are computed, followed by a second-order difference calculation. The sign of the second-order difference is used to determine the segmentation: a positive sign indicates increasing nonlinear error, at which point a counter is incremented by one. When the counter reaches or exceeds four, segmentation is performed starting from the first point; otherwise, no segmentation is conducted, and the counter is reset to zero.

Step 3: Based on the segmentation results, linear fitting is applied individually to each segment to establish regression models for the respective intervals. Furthermore, to reduce fitting errors near the segment boundaries and improve the continuity of the piecewise fitted curve, Hermite interpolation is employed to ensure smoothness.

The piecewise compensation model is expressed as in Equation (9):

(9) $$f\left(x\right)=\left\{\begin{array}{cc} \sum_{k=0}^{n}a_{k}x^{k} & \left(-x_{n}<x\leq -x_{n-1}\right)\\ \vdots & \\ \sum_{k=0}^{n}d_{k}x^{k} & \left(-x_{1}<x<x_{1}\right)\\ \vdots & \\ \sum_{k=0}^{n}g_{k}x^{k} & \left(x_{n-1}\leq x<x_{n}\right) \end{array}\right.$$

## 5. Experimental Verification

**Figure 8:**
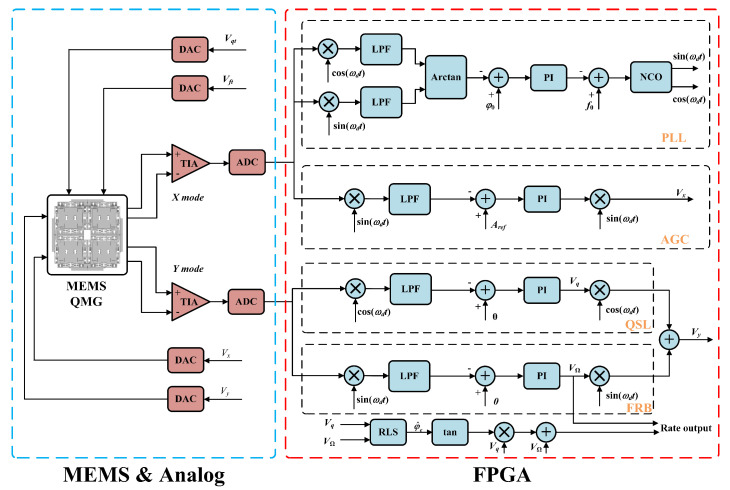
Closed-loop control block diagram of the MEMS QMG.

To verify the effectiveness of the proposed method, a closed-loop control block diagram of the QMG operating in force-to-rebalance (FTR) mode was established, as shown in Figure 8. The system includes the drive loop, orthogonal error suppression loop, and force-to-rebalance loop, with tuning performed in open loop. The drive closed-loop consists of a phase-locked loop (PLL) and automatic gain control (AGC). The AGC maintains the stable amplitude and frequency operation of the gyroscope drive mode, ensuring reliable sensing of angular rate. The PLL’s primary function is to rapidly track the resonant signal’s frequency and phase, providing two orthogonal phase demodulation and modulation reference signals.

Due to limitations in fabrication process and accuracy, stiffness coupling errors and frequency split inevitably occur in MEMS gyroscopes. Therefore, the orthogonal error suppression loop and open-loop tuning voltage are necessary to calibrate orthogonal errors and achieve modal matching [22,23], thereby improving gyroscope accuracy and signal-to-noise ratio (SNR). The force-to-rebalance closed-loop maintains the vibration displacement of the sensing mode at zero through feedback force, keeping the resonator vibration at a fixed position. The amplitude of the feedback force is used to obtain the gyroscope’s angular rate, with the magnitude of this additional force signal being proportional to the input angular rate [24], thus enabling demodulation from the force signal.

**Figure 9:**
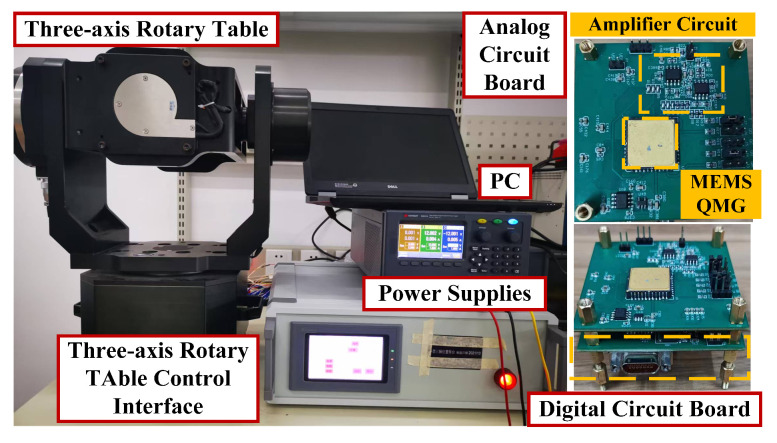
Scale-factor testing platform for the gyroscope.

Figure 9 shows the assembled MEMS QMG scale-factor testing system. The experimental setup consists of a high-precision three-axis turntable, a power supply, the gyroscope prototype, and a host computer. The high-precision turntable provides input angular velocities to the gyroscope prototype ranging from 0 to ±200°/s. The power supply delivers ±15 V and 5 V to the prototype, where ±15 V powers the analog board of the prototype and 5 V supplies the FPGA digital board. The gyroscope prototype comprises the die, analog board, and digital board. The analog board integrates the gyroscope die and signal detection circuits, which employ transimpedance amplifiers primarily based on the AD8642 operational amplifier. The digital circuitry consists of an FPGA, a 24-bit high-precision ADC, and a 16-bit DAC. The host computer is used to acquire and analyze the output data of the gyroscope prototype under different input angular velocities. The gyroscope prototype is mounted on the plane of the three-axis rate table using a customized fixture.

Under room temperature conditions, the scale-factor nonlinearity test of the MEMS QMG was conducted after powering the gyroscope and allowing a 1 min warm-up. The rate table was programmed to input a total of 22 angular rates: ±0.1°/s, ±0.2°/s, ±0.5°/s, ±1°/s, ±2°/s, ±5°/s, ±10°/s, ±20°/s, ±50°/s, ±100°/s, and ±200°/s. Simultaneously, the host computer was activated to record the gyroscope output data at each angular rate. For each angular rate, data were collected continuously for 2 min at a sampling rate of 100 Hz.

For comparative analysis, the gyroscope prototype was tested under three different conditions: (1) without any compensation applied to the scale factor, where raw scale-factor data were collected; (2) with polynomial fitting compensation (conventional method) [12], where scale-factor data after polynomial compensation were recorded; and (3) using the proposed composite compensation method, where scale-factor data after initial calibration combined with segmented compensation were obtained. Additionally, to verify the effectiveness of the proposed method in improving the repeatability error of the gyroscope scale factor, three repeated experiments were conducted under each condition.

**Figure 10:**
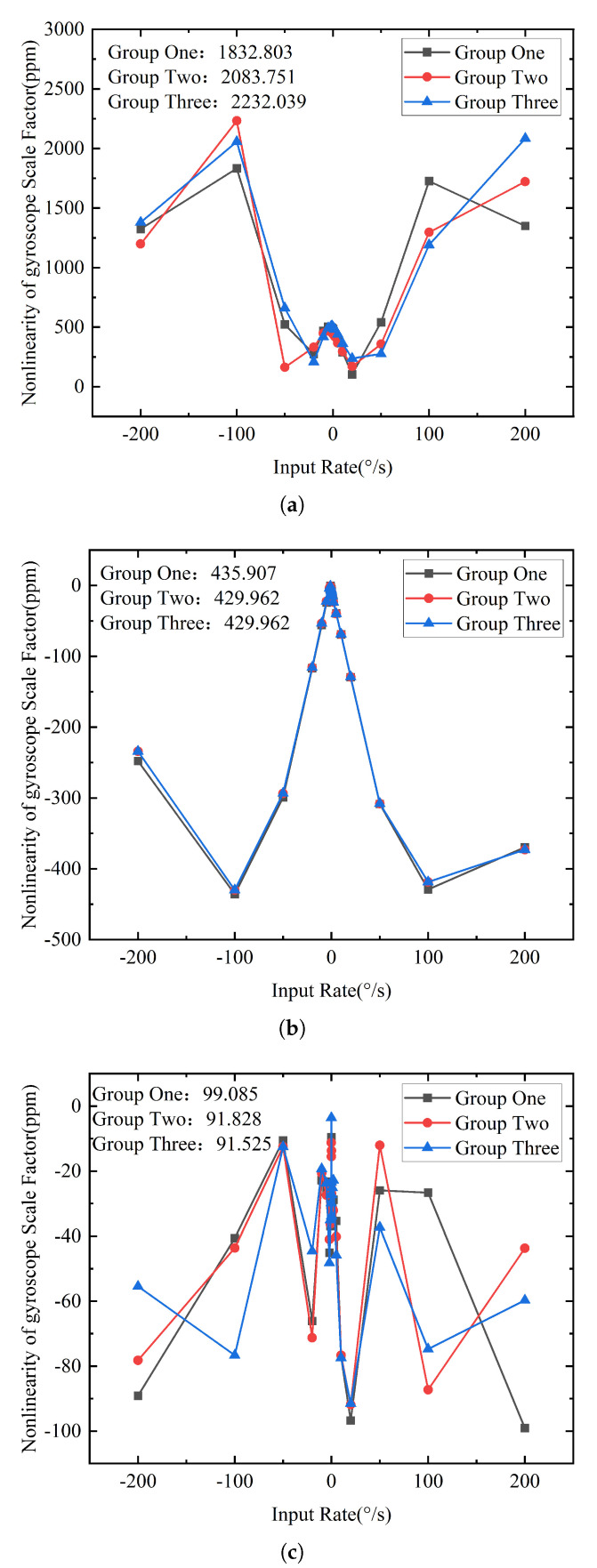
Comparison of scale-factor nonlinearity test results under three different methods. (**a**) Scale-factor nonlinearity test results without compensation. (**b**) Scale-factor nonlinearity test results under traditional polynomial compensation. (**c**) Scale-factor nonlinearity test results under the proposed method.

Figure 10 presents the scale-factor nonlinearity test results for the uncorrected case, the traditional polynomial fitting method, and the method proposed in this paper. The worst scale-factor nonlinearity without compensation reached 2232.039 ppm, with a scale factor of 13,709.853 LSB/°/s. As shown in Figure 10a, the repeatability error was relatively large, measuring 1230.656 ppm. Under the traditional polynomial fitting compensation, the worst scale-factor nonlinearity error decreased to 435.907 ppm, with a scale factor of −13,694.696 LSB/°/s. Although repeatability partially improved, inconsistencies at certain angular rate points remained, resulting in a repeatability error of 163.253 ppm. With the proposed method, the worst scale-factor nonlinearity error was further reduced to 99.085 ppm, and the scale factor was 13,719.534 LSB/°/s. The repeatability error was significantly improved, decreasing to 93.534 ppm.

The calculation method for the repeatability error in this study is as follows: three sets of repeated experiments were conducted under the same compensation method; the scale factor was calculated based on the measurement results from each group; then, the standard deviation of the scale factors obtained from the three tests was computed; this value is defined as the repeatability error.

It is noteworthy that the sign of the scale factor obtained in Figure 10b differs from those in Figure 10a,c, which is attributed to the inconsistency between the forward and reverse rotation directions set by the rate table itself. Furthermore, due to the longer time required for temperature stabilization during the first set of repeated experiments under the same prototype condition, discrepancies were observed between the first set and the subsequent second and third sets. This temperature-related variation is difficult to estimate and mitigate.

Comparing the scale-factor nonlinearity test results under the three conditions, the proposed method reduces the gyroscope’s closed-loop scale-factor nonlinearity error by 22.5 (2232.09 ppm/99.085 ppm) times and 4.4 (435.907 ppm/99.085 ppm) times compared to the original data and traditional compensation method, respectively; the repeatability error is reduced by 13.1 (1230.656 ppm/93.534 ppm) times and 1.7 (163.253 ppm/93.534 ppm) times, respectively. These results demonstrate the effectiveness of the proposed composite compensation method with initial calibration for MEMS gyroscope scale-factor nonlinearity. Moreover, it improves both the nonlinearity and repeatability of the gyroscope’s closed-loop scale factor, providing a reliable guarantee for the dynamic performance of high-precision MEMS gyroscopes. In addition, to provide a more intuitive comparison of the differences among the three test results, all data are summarized in Table 3.

## 6. Conclusions

The scale factor of MEMS gyroscopes exhibits significant nonlinear dependence on the input angular rate, particularly under high-rate conditions where nonlinearity becomes more pronounced. Additionally, scale-factor repeatability, affected by parameter drift, has emerged as a key limitation to effective nonlinear compensation. To address these challenges, this study presents a compensation strategy for scale-factor nonlinearity in a MEMS gyroscope prototype. First, the underlying mechanism of scale-factor nonlinearity and the factors limiting current compensation performance are analyzed. A composite compensation method incorporating initial calibration is then proposed to mitigate repeatability errors through accurate baseline scale-factor correction. Building on this, a piecewise polynomial fitting approach is introduced to jointly compensate for both repeatability and nonlinear scale-factor errors in the closed-loop system. Experimental results show that, compared with the uncompensated case, the proposed method improves scale-factor repeatability and nonlinearity by factors of 13.1 and 22.5, respectively, confirming its accuracy and practical applicability. This approach offers an effective solution for mitigating scale-factor nonlinearity in MEMS gyroscopes and improving their dynamic performance.

Nevertheless, the critical challenge of temperature variation causes the proposed composite closed-loop scale-factor compensation method for MEMS gyroscopes to experience zero-rate output drift under rapid temperature changes. This limits its effectiveness in mitigating scale-factor nonlinearity and repeatability errors, a well-known issue inherent to MEMS sensors. Consequently, future work will focus on (1) developing real-time scale-factor compensation techniques that are effective over the entire temperature range and (2) exploring force-rebalanced closed-loop control approaches distinct from conventional AC-to-DC conversion structures to mitigate their impact on scale-factor performance.

## Figures and Tables

**Table 1:** Mechanical parameters of the MEMS QMG.

Parameters	Value
Mass	1.56 mg
Frequency (X)	4634 Hz
Frequency (Y)	4711 Hz
Quality factor (X)	$1.02\times 10^{6}$
Quality factor (Y)	$0.34\times 10^{6}$
Initial frequency split	77 Hz
Velocity to current factor (X)	$2.41\times 10^{-6}$ A/m/s
Voltage to force factor (X)	$2.79\times 10^{-9}$ N/V
Velocity to current factor (Y)	$6.61\times 10^{-6}$ A/m/s
Voltage to force factor (Y)	$4.52\times 10^{-9}$ N/V

**Table 2:** Nonlinearity experiment results of DAC output with AC signal.

Input Rate (°/s)	${\mathrm{DA}}_{\mathrm{word}}$	$V_{a}c$/V
−200	29E345	1.637
−100	14F191	0.816
−50	A78B6	0.408
−20	43,033	0.163
−10	21,808	0.0815
−5	10BF2	0.0407
−2	6B19	0.0163
−1	357B	0.00816
−0.5	1AAB	0.00408
−0.2	A95	0.00163
−0.1	539	0.0008217
0.1	FFA81	−0.000809
0.2	FFF524	−0.00162
0.5	FFE50E	−0.00407
1	FFCAF3	−0.00815
2	FF94A1	−0.0163
5	FEF3C7	−0.0408
10	FDE7B1	−0.0816
20	FBC786	−0.163
50	F58F03	−0.408
100	EB0E29	−0.816
200	D61C74	−1.637

**Table 3:** Test results of scale-factor errors under three conditions.

Group	Origin	Tra Method	This Work
One (ppm)	1832.803	435.907	99.085
Two (ppm)	2083.751	429.962	91.828
Three (ppm)	2232.039	429.962	91.525
SF (LSB/°/s)	13,709.853	−13,694.696	13,719.534
Repetitive error (ppm)	1230.656	163.253	93.534

## Data Availability

The data presented in this study are available upon request from the corresponding author. The data are not publicly available due to privacy.
